# The P23H Rhodopsin Mouse Model Reveals a Novel Interaction Between the Endoplasmic Reticulum and Connecting Cilium Rootlet Within Photoreceptors

**DOI:** 10.1167/iovs.67.3.57

**Published:** 2026-03-30

**Authors:** Sergey S. Novoselov, Bernardo S. Mendes, Andrea Martello, Silene Wavre-Shapton, Monica Aguila, Rosellina Guarascio, Kalliopi Ziaka, Dalila Bevilacqua, Gergana Metodieva, Philip J. Luthert, Jun Yang, Metodi V. Metodiev, Philip J. Reeves, Clare E. Futter, Michael E. Cheetham, Thomas Burgoyne

**Affiliations:** 1 University College of London Institute of Ophthalmology, London, United Kingdom; 2 Moorfields Eye Hospital, London, United Kingdom; 3 School of Life Sciences, University of Essex, Colchester, United Kingdom; 4 Department of Ophthalmology and Visual Sciences, Moran Eye Center, University of Utah, Salt Lake City, Utah, United States; 5 Division of Otolaryngology, Department of Surgery, University of Utah, Salt Lake City, Utah, United States; 6 Department of Neurobiology, University of Utah, Salt Lake City, Utah, United States

**Keywords:** membrane contact sites, rootlet, mitochondria, endoplasmic reticulum (ER), rhodopsin

## Abstract

**Purpose:**

Photoreceptors are highly polarized sensory neurons containing a modified cilium known as the outer segment. This cilium has a rootlet that spans the length of the metabolically active inner segment and anchors the outer segment to the remainder of the photoreceptor. The full function and reasons for such a long rootlet in photoreceptors are not well understood. To gain deeper insight, we characterized the membrane associated with the rootlet.

**Methods:**

Proteomic analysis was performed on immunopurified wild-type (WT) and P23H rhodopsin knock-in mouse retina. Images of mouse and human retina were acquired by transmission electron microscopy and electron tomography and protein localization in mouse retina determined by immunofluorescence and immunoelectron microscopy.

**Results:**

In homozygous P23H knock-in mouse retinas, misfolded rhodopsin retained in the endoplasmic reticulum (ER) prior to degradation was found to be closely associated with rootletin and mitochondrial proteins. This observation helped reveal that the ER forms extensive interactions with the rootlet, running alongside it throughout the inner segment. Furthermore, the ER branches from the rootlet to make contact with mitochondria, Golgi, and the plasma membrane. Human rod photoreceptors had similar rootlet:ER interactions within the proximal inner segment, but differed from mouse, as the rootlet within the distal inner segment mainly interacted with mitochondria.

**Conclusions:**

These findings suggest that the rootlet plays a critical role in organizing intracellular architecture by serving as a kind of “scaffold” that supports ER positioning and allowing it to branch and form membrane contact sites with other cellular membranes.

Photoreceptors are highly specialized neurons that enable vision via a modified sensory cilium known as the outer segment.^1^^–^^3^ The outer segment consists of stacked disc membranes that house the phototransduction machinery that detects light. The outer segment is anchored to the inner segment by the connecting cilium. At the basal body and base of the connecting cilium, a cytoskeleton like rootlet is attached that extends through the inner segment. The rootlet is a striated structure comprising homopolymeric rootletin protofilaments bundled together to form thick filaments.^4^

The rod photoreceptor inner segment contains the endoplasmic reticulum (ER) and Golgi where outer segment membrane proteins are synthesized and processed before traffic to the base of the cilium and subsequent transport to the outer segment via the ciliary plasma membrane.^5^^–^^7^ The outer segment is subject to photo-oxidative damage and so must be replaced in its entirety approximately every 10 days meaning that traffic of proteins via the cilium occurs in unparalleled quantity. The importance of this transport is exemplified by the retinal degeneration associated with pathogenic variants in rhodopsin (*RHO*), which encodes the light sensitive protein of rods and forms >50% of rod outer segment protein. The P23H amino acid substitution in RHO is the most common cause of autosomal dominant retinitis pigmentosa (ADRP) in the United States. ADRP is characterized by photoreceptor death and loss of peripheral vision.^8^^–^^10^ The P23H substitution leads to protein misfolding, ER retention, and degradation.^11^^–^^13^

There is evidence that the rootlet plays a role in centrosome cohesion and positioning.^14^^–^^17^ In photoreceptors, the connecting cilium rootlet is much larger than in other ciliated cell types and extends 50 µm or more from the proximal inner segment all the way to the synaptic terminal.^18^ In addition to anchoring the outer segment to the inner segment, it has been proposed to assist in the transport of proteins to the outer segment for the formation of new discs.^19^^,^^20^ When ablated in rootletin mutant mice, an apparently normal connecting cilium and outer segment were formed initially, but with time there was shortening and loss of outer segments, and photoreceptor cell death.^21^ The connection between the rootlet and the biosynthetic or transport machinery is unclear, but membranes adjacent to the connecting cilium rootlet have been previously described.^4^^,^^22^ Interactions between neighboring organelles are mediated by membrane contact sites (MCS), regions where the membranes of adjacent organelles come within 30 nm of each other and are maintained by tethers. These MCS play a major role in organelle function by mediating, for example, protein:protein interactions, lipid transport, and ion exchange. However, the rootlet lacks a membrane and so it is not clear how it interacts with neighboring membranes.

In this study, analysis of the interactome of P23H rhodopsin that is retained in the ER surprisingly identified rootletin as a major putative interacting protein. We identify the membranes that associate with the rootlet as ER, where rhodopsin is retained in heterozygous P23H mice. We demonstrate, in control mice, using three-dimensional (3D) electron microscopy that the ER branches from the rootlet to make extensive MCS with other cellular membranes. Differences in the organelle interactions of the rootlet between mouse and human photoreceptors were identified. These data support a role for the long rootlet within photoreceptors in facilitating organelle interactions in this highly polarized cell and highlight important key differences between species.

## Methods

### Human Tissue

Human donor eyes were collected from a 43, 77, and a 90-year-old cadaver with no known eye disease at the John A. Moran Eye Center, University of Utah, Salt Lake City, Utah, USA, between 2007 and 2020 in collaboration with local hospitals and eye banks and were held at the Sharon Eccles Steele Center for Translational Medicine, John A. Moran Eye Center. All participants were willing eye donors, and written informed consent was obtained from surviving relatives. The study adhered to the Declaration of Helsinki and was approved by the Institutional Review Boards of the University of Utah.

### Mouse Tissue

P23H rhodopsin (Rho^P23H^) knock-in mice on a C57Bl6J background were a gift from Professor K. Palczewski and were generated as previously described.^9^ Animals were bred at the University College of London (UCL) Institute of Ophthalmology with Institutional ethical and under a UK Home Office approval.^9^^,^^23^ Wild type C57Bl6J and Rho(P23H) knock-in mice were euthanized in accordance with Animals (Scientific Procedures) Act 1986 (United Kingdom) and Home Office (United Kingdom) guidance rules, adhering to the Association for Research in Vision and Ophthalmology Statement for the Use of Animals in Ophthalmic and Vision Research.

### Immunoprecipitation

Dissections of post-natal day 10 (P10) mouse retinas were performed in ice cold PBS buffer with immediate transfer of 4 pooled samples per genotype (homozygous P23H-knock-in or wild-type [WT] littermates) into 450 µL of 1% n-dodecyl-beta-D-maltoside (DM; Sigma) lysis buffer in PBS containing 5% protease and phosphatase inhibitor cocktail (Halt, Pierce). Lysis was further encouraged by pulsed sonication on ice for 10 seconds (QSonica) followed by preclearing at 21,300*g* in a bench-top centrifuge for 1 minute. Supernatant was supplemented with 30 µL of pre-washed Protein-G Dynabeads (Invitrogen) and 3 µL of 1D4 antibody (Abcam) per sample and incubated overnight at +4°C. Magnetically separated Dynabeads were then washed 5 times with 1 mL of PBS containing 0.025% of Tween-20 (Sigma) and sequential elution was performed with 250 µM 1D4 peptide (TETSQVAPA), 0.5 M NaCl in PBS and 1 × Laemmli buffer with 3 minutes of incubation at 98°C. Eluates were then snap-frozen in liquid nitrogen and used for mass spectrometry analysis.

### Mass Spectrometry

Protein samples were digested with trypsin, as described in Reference 24 and analyzed by nano-scale liquid-chromatography tandem mass spectrometry (LC-MS/MS) to generate raw datafiles. A high-resolution hybrid LTQ/Orbitrap Velos instrument was used. The mass spectrometer was operated in data-dependent mode, as described in Reference 24. Briefly, a 90-minute gradient was used to separate the tryptic peptide mixtures. The instrument executed a full scan followed by 20 MS/MS scans to isolate and sequence the 20 most abundant precursor ions. The generated raw files were analyzed by MaxQuant as described in Reference 24. Proteins filtered for those identified by <2 peptides and immunoglobulins and trypsin were removed from the list (Supplementary Table S1) and CRAPome scores were generate using the online tool (https://reprint-apms.org/).^25^

### Immunofluorescence

Mouse eyes were fixed after extraction in 4% paraformaldehyde (PFA) in PBS for 1 hour at room temperature before infusing with 30% sucrose in PBS overnight at 4°C. The eyes were embedded in OCT compound and 14 µm cryostat sections cut. Sections were permeabilized with 0.2% saponin in PBS for 20 minutes before incubating in blocking solution containing 0.02% saponin, and 1% BSA in PBS for 30 minutes at room temperature. Antibodies against Mitofusin 1 (1:200, sc-166644; Santa Cruz), Rootletin,^4^ Protein disulfide-isomerase (1:100, GTX101468, Insight Biotechnology), and KDEL (1:100, NBP1-97469, Novus Biologicals) were added for 1 hour in blocking solution at room temperature. Secondary antibodies donkey anti-mouse IgG Alexa Fluor 488 (1:250, A21202, Thermo Fisher Scientific), donkey anti-mouse IgG Alexa Fluor 555 (1:250, A31570, Thermo Fisher Scientific), donkey anti-rabbit IgG Alexa Fluor 488 (1:250, A21206, Thermo Fisher Scientific), donkey anti-rabbit IgG Alexa Fluor 555 (1:250, A31572, Thermo Fisher Scientific), and goat anti-chicken IgY Alexa Fluor 555 (1:250, A21437, Thermo Fisher Scientific), were applied in blocking solution to samples for 1 hour at room temperature.

### Transmission Electron Microscopy

Whole mouse eyes and pieces of human eye tissue were fixed in 2% PFA, 2% glutaraldehyde in 0.1 M cacodylate buffer, and pH 7.4 for 1 hour at room temperature. The cornea and lens were removed from the mouse eyes, and these were fixed for a further 1 hour at room temperature. All samples were washed in cacodylate buffer before being incubated in 1% osmium tetroxide, 1.5% potassium ferrocyanide in distilled water for 1 hour in the dark at 4°C. Subsequently, the samples were dehydrated in increasing concentrations of ethanol (70%, 90%, and 100%) and in a mixture of propylene oxide:epon (1:1) overnight at room temperature. The propylene oxide:epon was removed and replaced with two changes of epon at room temperature before embedding in epon overnight at 60°C. Then, 100 nm sections were cut and imaged on a JEOL 1400Plus EM fitted with both an Advanced Microscopy Technologies (AMT) NanoSprint 12 and a Gatan Orius SC1000B camera.

### Electron Tomography

Tilt series were collected on a JEOL 1400Plus setup with the software SerialEM (developed at University of Colorado, Boulder, CO). Images were collected over a range of ±60 degrees with 2 perpendicular axes, and dual-axis tomograms were generated from the tilt data using IMOD (developed at the University of Colorado, Boulder, CO).^26^ Image data was viewed in 3dmod (part of the IMOD package) and ImageJ software.

### ImmunoElectron Microscopy

Mouse eyes were fixed in 4% PFA and 0.1% glutaraldehyde in 0.1 M phosphate buffer at pH 7.4 for 1 hour, before removing the cornea and lens and fixing for a further 1 hour. The eye cups were cut into small blocks and embedded in 12% gelatin, followed by infusion with 2.3 M sucrose solution at 4°C overnight. Then, 80 nm sections were cut at −120°C and collected in a 1:1 mixture of 2.3 M sucrose/2% methylcellulose, and labeling was performed. Single labeling with anti-KDEL (1:10, NBP1-97469, Novus Biologicals) and double labeling using two anti-Rhodopsin antibodies RET-P1 (1:100, Abcam) and 1D4 (1:250) was performed using methods previously described.^27^ Quantitation of immuno-gold labeling was undertaken using the Gold Particle Analyser program.^28^ With this program, gold particles were automatically detected across whole images, and the draw polygon tool was used to define a region surrounding the plasma membrane of cross-sectionally oriented photoreceptor inner segments or within 100 nm of the rootlet. The software provided measurements of both the polygon area and the number of gold particles contained within this region. In longitudinally oriented inner segments, rootlet length was measured using the line tool. Due to ultrathin 100-nm thick transmission electron microscopy (TEM) sections being used, only regions of the rootlets were visible. Therefore, to account for differences in rootlet length, the number of gold particles per unit length of rootlet was calculated.

### Serial Block Face Scanning Electron Microscopy

Eyes were fixed in 3% glutaraldehyde and 1% PFA in 0.08 M sodium cacodylate buffer, pH 7.4 for 2 hours at room temperature and prepared as previously described.^29^ Using a Gatan 3View system (Gatan Inc, Abingdon, UK) and a Zeiss Sigma VP field emission scanning electron microscope (Zeiss, Cambridge, UK), images were acquired in between the sequential cutting away of a 100-nm thick section of the sample. The images were re-aligned using the StackReg plugin (EPFL) in ImageJ software (NIH) and segmentation of the data was done using the 3dmod (part of the IMOD tomography package)^26^ and Microscope Image Browser (MIB developed at University of Helsinki). The segmentation was visualized in Chimera (UCSF).

## Results

### Misfolded P23H Rhodopsin Provides Evidence That the ER Is Associated With the Rootlet and has Membrane Contact Sites With Mitochondria

**Figure 1.**
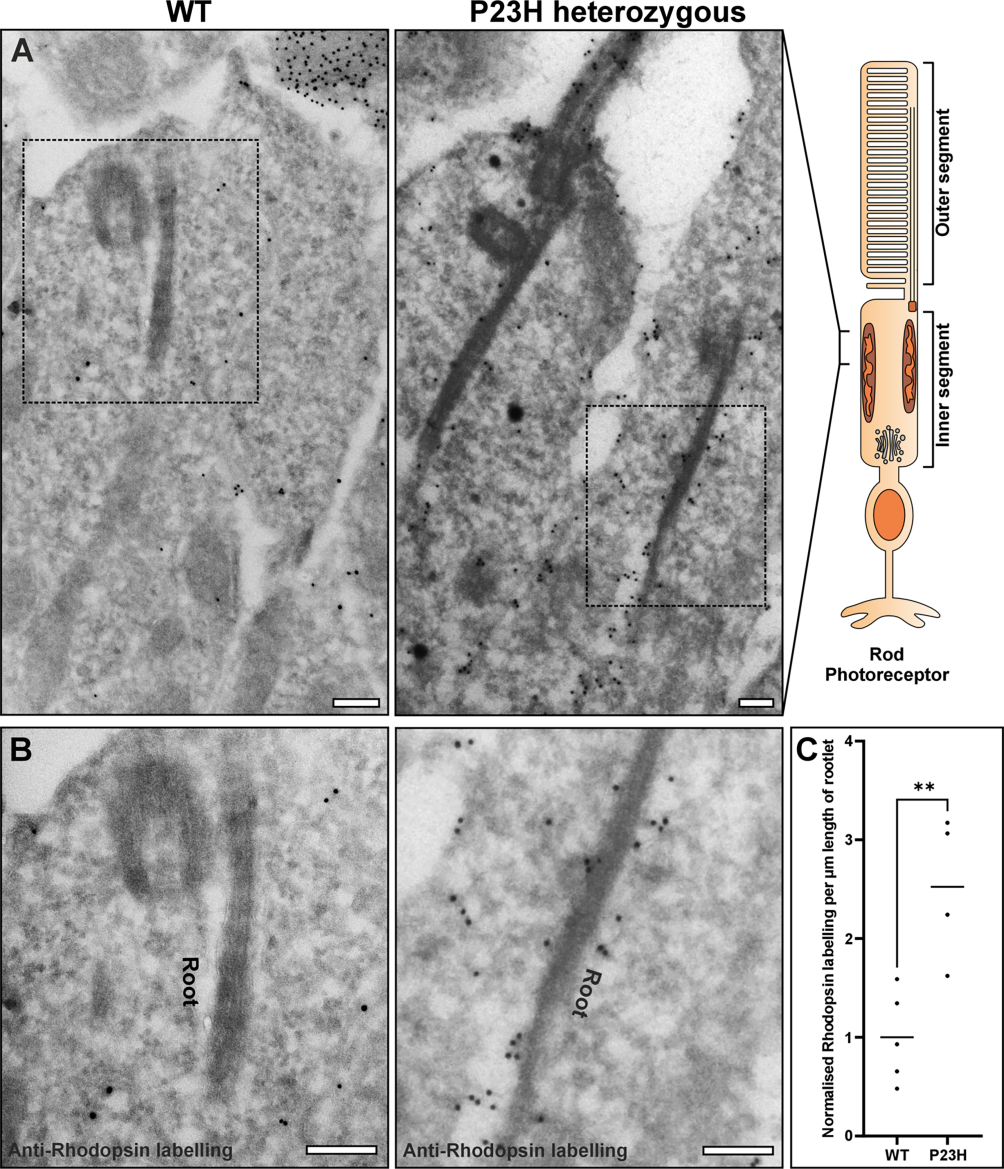
**Rhodopsin** **localization** **near the rootlet in P23H mice**. (**A,** **B**) ImmunoEM labelling for rhodopsin in 6-week-old WT and heterozygous P23H mouse retina. The *dashed box* shows the region zoomed in focusing on the rootlet in **B**. **B** ImmunoEM labeling for rhodopsin show little staining in WT mouse, whereas heterozygous P23H mouse retina has much more staining close to the rootlet, identifiable by its striated appearance. (**C**) Measurement of the number of immunoEM gold particles associated with rhodopsin staining within 100 nm of the rootlet. Electron microscopy sections (5 for WT and 4 for P23H) were immuno-labeled in 2 batches, and ≥5 rootlets per section were analyzed. The results were normalized between staining batches to the mean of number of gold particles within 100 nm of the rootlet in WT mice. Plot shows the mean and statistical significance determine using an unpaired, two-tailed Student's *t*-tests: **P ≤ 0.01. *Scale bar* = 200 nm.

The ADRP P23H rhodopsin knock-in mouse model is known to accumulate misfolded rhodopsin within the ER after protein synthesis, prior to degradation.^10^ Labeling for rhodopsin by immunoelectron microscopy (immunoEM), clearly shows rhodopsin immunoreactivity within the ER that appears to be associated with the photoreceptor inner segment rootlet of the P23H heterozygous mouse (Fig. 1; Supplementary Fig. S1). The rootlet is identifiable as a long tubular-like striated structure that runs through the inner segment.

To identify proteins that associate with P23H rhodopsin, mouse retinas from homozygous P23H and WT C57Bl6J retinas at P10 were lysed in DM buffer and immunopurified with the anti-rhodopsin C-termini 1D4 antibody. This immunoaffinity method is widely used to purify functional rhodopsin and was modified with a magnetic bead immunopurification strategy that we had previously used to identify chaperone: aggregation prone protein interactions to avoid co-sedimentation of aggregated and insoluble proteins.^30^^,^^31^ To further reduce the potential for contamination with nonspecific binding proteins, the purified proteins were eluted sequentially with buffer containing 1D4 peptide, high salt, or SDS. Mass spectrometry of the eluates by nano-scale LC-MS/MS was used to identify potential interacting peptides (see Supplementary Table S1). In total, over 500 proteins were identified by at least one peptide spectral count in all 3 elution conditions, but this was reduced after filtering for proteins identified by at least 2 different peptide spectral counts. Importantly, most of the rhodopsin was eluted from the WT retina by the most stringent elution with the 1D4 peptide, so this condition was used for the subsequent analyses of putative interactors. Following these filtering steps, 99 proteins were identified from the P23H mouse retina, whereas 62 proteins were identified from the WT retina. These proteins were cross-referenced against the “crapome” database,^25^ which lists proteins that are commonly found in mass spectroscopy-based interaction experiments, potentially as nonspecific contaminants. The top 20 proteins that do not commonly occur in other databases (less than 1 in 3) are shown in the Table. As expected, the most abundant peptides in the WT retina corresponded to rhodopsin, with slightly lower level of rhodopsin peptides from P23H retina, possibly related to the degradation or aggregation of P23H rhodopsin.

**Table.** Rootletin In The P23h Interactome

Protein Names	Gene Name	Organelle and/or Function	WT 1D4 Elution Peptides Measured	P23H 1D4 Elution Peptides Measured	Fold Enrichment in P23H
Rootletin	*Crocc*	Rootlet	0.5 (± 0.71)	62 (± 0.00)	124
**Rhodopsin**	***Rho***	Inner/outer segment	79.5 (± 12.02)	58 (± 4.24)	0.73
Drebrin	*Dbn1*	Cytoskeleton	0.5 (± 0.71)	14.5 (± 3.54)	29
**Alpha-(1,6)-fucosyltransferase**	***Fut8***	Golgi apparatus	0 (± 0.00)	12.5 (± 6.36)	P23H only
F-actin-capping protein subunit alpha-2	*Capza2*	Cytoskeleton	1.5 (± 2.12)	11 (± 1.41)	7.33
**Non-selective voltage-gated ion channel VDAC1**	***Vdac1***	Mitochondria	2.5 (± 0.71)	8 (± 1.41)	3.2
**Phosducin**	***Pdc***	Inner/outer segment	1.5 (± 0.71)	6 (± 0.00)	4
**Cytochrome b-c1 complex subunit 2**	***Uqcrc2***	Mitochondria	1 (± 0.00)	5.5 (± 2.12)	5.5
Tropomodulin 2	*Tmod2*	Cytoskeleton	0 (± 0.00)	5.5 (± 0.71)	P23H only
**Protein sel-1 homolog 1**	***Sel1l***	ER	0.5 (± 0.71)	5 (± 1.41)	10
Large ribosomal subunit protein eL43	*Rpl37a*	Ribosomal	3 (± 2.83)	4.5 (± 3.54)	1.5
**ATP synthase peripheral stalk subunit OSCP**	***Atp5o***	Mitochondria	4 (± 0.00)	3 (± 0.00)	0.75
**Protein OS-9**	***Os9***	ER	0 (± 0.00)	3 (± 0.00)	P23H only
NADH-ubiquinone oxidoreductase 75 kDa subunit	*Ndufs1*	Mitochondria	0 (± 0.00)	3 (± 1.41)	P23H only
**Myelin expression factor 2**	***Myef2***	Nucleus	0 (± 0.00)	2.5 (± 0.71)	P23H only
**Unconventional myosin-VI**	***Myo6***	Cytoskeleton	0 (± 0.00)	2.5 (± 0.71)	P23H only
**Serine/threonine-protein kinase ATR**	***Atr***	Nucleus	1 (± 0.00)	2 (± 0.00)	2
**Sodium/potassium-transporting ATPase subunit beta-2**	***Atp1b2***	Plasma membrane	3.5 (± 0.71)	2 (± 1.41)	0.57
**MICOS complex subunit Mic60**	***Immt***	Mitochondria	0 (± 0.00)	2 (± 1.41)	P23H only
Glutathione S-transferase Mu 3	*Gstm3*	Cytoplasm	2 (± 0.00)	2 (± 0.00)	1

Top 20 proteins with the highest peptide spectral count that were immunopurified and eluted with 1D4 peptide from P10 WT and P23H rhodopsin homozygous mouse retina. Mean ± SD of spectral counts measured from two independent purifications.

Proteins in bold were also identified in a previous study that examined the rhodopsin interactome in P23H rhodopsin homozygous mouse retina.^32^ Proteins with a high CRAPome score are not included in the table.^25^

The putative rhodopsin interactome was compared to a previous interactome study from P15 homozygous P23H mouse retina, which used a similar purification method but with elution in 8 M urea.^32^ There was a high degree of concordance, with 66% of the proteins identified here previously reported to interact with WT or P23H rhodopsin (see Supplementary Table S1).^32^ The highest degree of agreement was in the chaperones and ER quality control components (Supplementary Table S2), mitochondrial proteins (Supplementary Table S3) and ribosomal proteins (Supplementary Table S4), which were mainly enriched in the P23H immunopurification. Strikingly, many of the novel putative interacting proteins were associated with the cytoskeleton. In particular, rootletin was the most highly enriched protein from P23H mice, but was only a minor component of those from WT mice (see the Table; Supplementary Table S5). Whereas several of the cytoskeletal proteins are found frequently in other interactome datasets, rootletin has only been reported in 36 from 716 experiments. Most of the mitochondrial proteins immunopurified were also enriched in the P23H purifications and may reflect interactions that occur at MCS between the ER and mitochondria within the inner segment (see Supplementary Tables S2, S3).^32^

### 3D Electron Microscopy and Immuno-Labeling Show the ER Is Associated With the Rootlet

**Figure 2.**
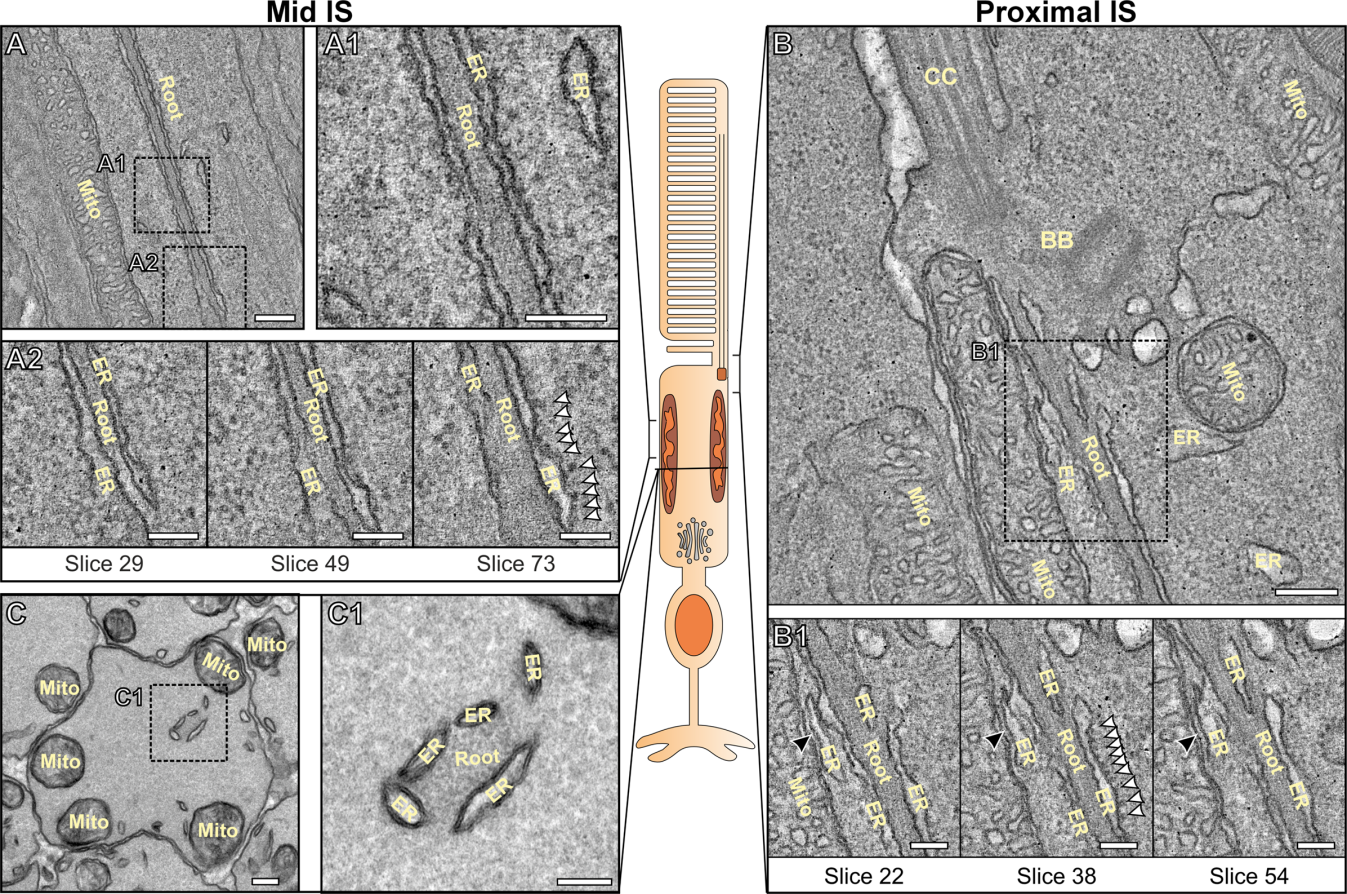
**Membranes associated with the photoreceptor rootlet**. (**A,** **B**) Tomograms of 6-month-old control mouse retina showing the rootlet (Root) taken **A** toward the center of the inner segment and **B** at the distal region of the rod inner segment where the connecting cilium (CC) and basal bodies (BB) can be observed (A2 and B1). Slices from the tomograms show the membranes in contact with the rootlet have ribosome-like structures associated with them (*white arrowheads*) (A2 slice 73 and B1 slice 38). These membranes also extend and make contact with mitochondria (Mito) as shown by the *black arrowheads* in the proximal IS (B1 slice 22 and slice 54). (**C**) Transversely orientated electron microscopy sections of photoreceptors show the rootlet can be seen surrounded by ER like membranes. *Scale bars* = **A**, **B**, and **C** left panel = 200 nm and **A1**, **A2**, **B1**, and **C** right panel = 100 nm.

To further examine the rootlet of the connecting cilium of mouse rod photoreceptors we used 2D and 3D TEM techniques (Fig. 2; Supplementary Fig. S2). In electron tomography of WT mouse retina, the rootlet has characteristic filaments running throughout in longitudinally orientated samples (see Figs. 2A, 2B; Supplementary Fig. S3), and a punctate pattern of the filament bundles in cross-sectionally orientated samples (see Fig. 2C; Supplementary Fig. S4).^4^ From both longitudinal and cross section views, extensive membranes are visible running alongside and in contact with the rootlet. These membranes appear to encircle (see Fig. 2C) and run along the length of the rootlets (see Figs. 2A, 2B). From tomograms generated of the mid (see Fig. 2A) and distal (see Fig. 2B) regions of the inner segment, these membranes appear to be ER, as ribosome-like structures are visible on the side of the membrane facing away from the rootlet. In some regions, these ER-like membranes extend and branch away from the rootlet to make contact with mitochondria (see Fig. 2B1). These membranes have a tubular or sheet-like appearance often with ribosome-like associated structures that correspond with previous studies and descriptions of ER when imaged by electron microscopy.^33^^–^^35^ Similar membranes associated with the rootlet could be seen within cone photoreceptors (Supplementary Fig. S5).

**Figure 3.**
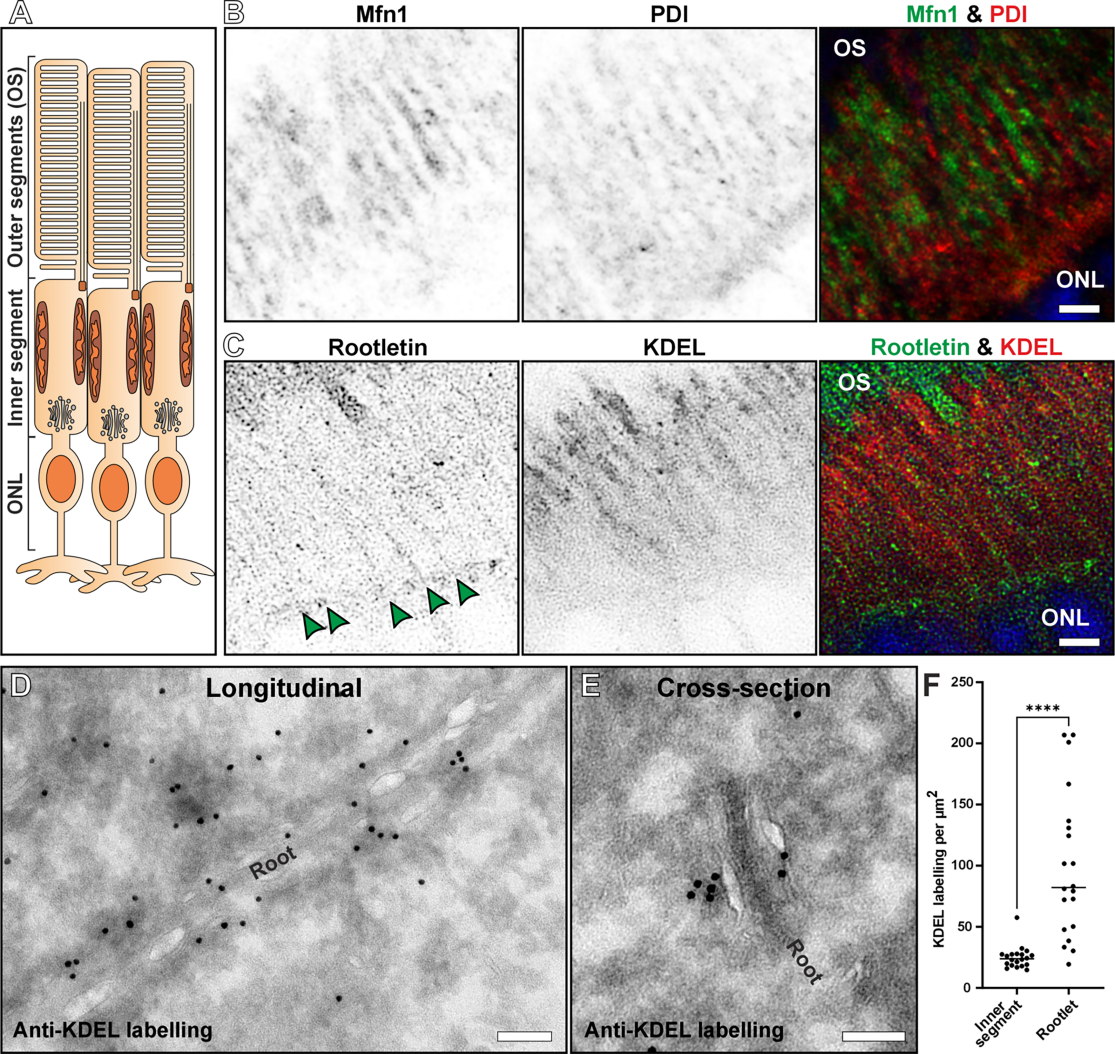
**ER association with the rootlet and mitochondria**. ER associations demonstrated by immunofluorescence and immunoelectron microscopy staining in 6-month-old control mouse retina. (**A**) Schematic diagram showing the inner segment region imaged in (**B,** **C**) between the outer segments (OS) and outer nuclear layer (ONL). **B** Immunoreactivity for the ER marker protein disulfide-isomerase (PDI) was observed running through the inner segment between the mitochondria stained with mitofusin 1 (Mfn1). **C** ER stained with anti-KDEL shows a close association with the rootlet marker rootletin. *Green arrowheads* point to the rootletin staining running through the photoreceptor inner segments. (**D,** **E**) The association of KDEL and the rootlet (Root) was also shown by immuno-EM labeling of KDEL. **D** Longitudinal and **E** transverse orientated inner segments show KDEL labeling on the membrane surrounding the rootlet. (**F**) The concentration of KDEL labeling is much higher within 100 nm of the rootlet compared with the entire cross-sectionally orientated inner segments. Plot shows the mean and statistical significance determine using a paired, two-tailed Student's *t*-tests: ****P ≤ 0.0001. *Scale bars* = **B** and **C** = 2.5 µm and **D** and **E** = 100 nm.

To determine if the rootlet associated membranes are ER, the localization of these organelles was examined by immunofluorescence and immunoEM (Fig. 3; Supplementary Fig. S6). Co-staining with the ER marker, protein disulfide isomerase (PDI), and the mitochondrial marker, Mitofusin 1, the ER appeared to run in between mitochondria within the inner segment (see Fig. 3B). As mitochondria are known to be positioned alongside the inner segment plasma membrane in mice,^29^ this staining indicates that the ER runs down through and close to the center of the inner segment. Staining for ER (anti-KDEL) and the rootlet (anti-rootletin) revealed a close association (see Fig. 3C). ImmunoEM labeling for the ER marker KDEL revealed strong staining of the membranes associated with the rootlet in both longitudinal and transverse oriented samples (see Fig. 3D; Supplementary Fig. S6). Quantification of the gold labeling density showed enrichment of KDEL close to the rootlet when compared to staining across whole inner segment cross sections (see Fig. 3F).

### The Photoreceptor ER Makes MCS With Other Organelles and the Plasma Membrane

**Figure 4.**
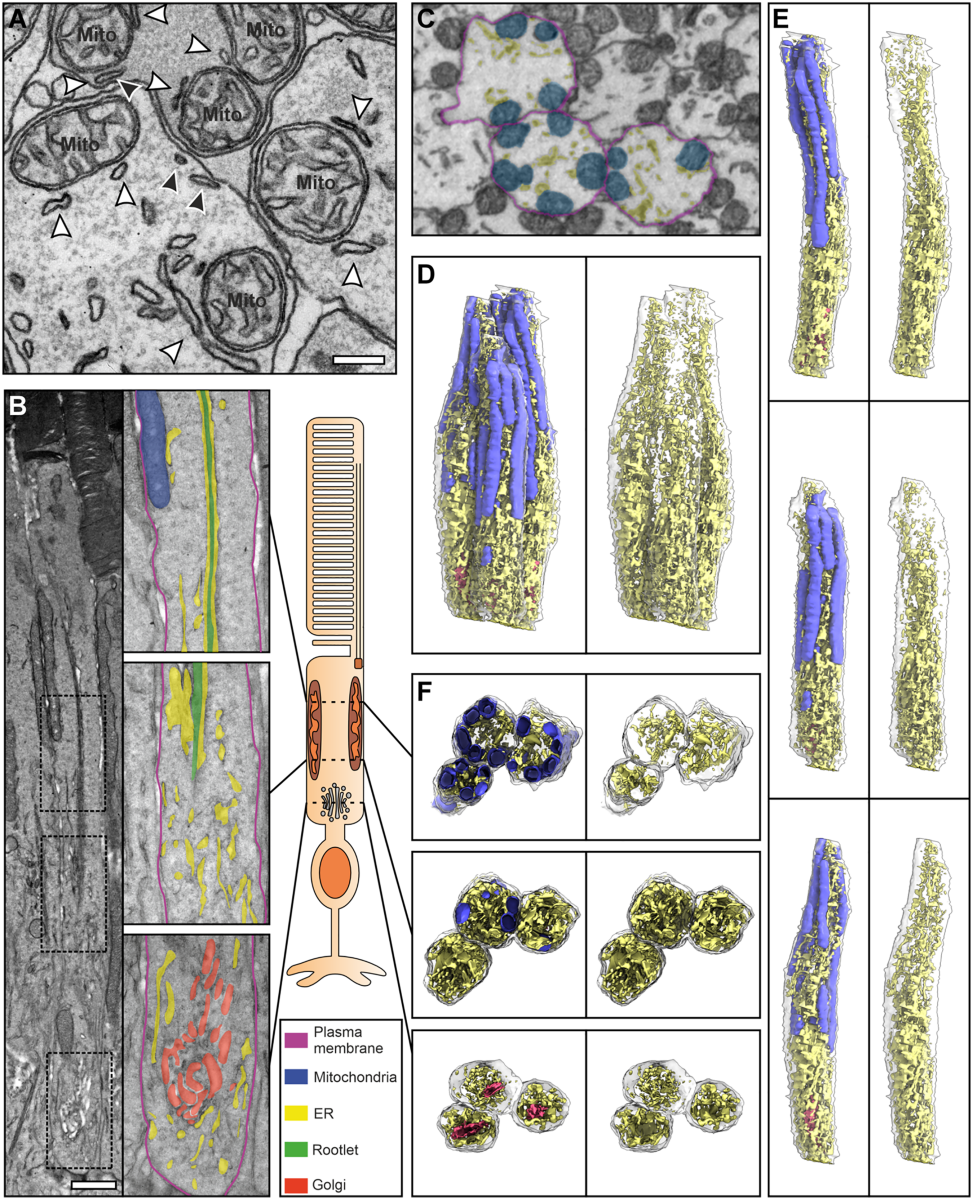
**Multiple contacts of the photoreceptor ER**. (**A**) Transverse view of control 6-month-old mouse photoreceptor inner segments, where the ER has MCS with mitochondria (*white arrowheads*) and the plasma membrane (*black arrowheads*). (**B**) Longitudinal orientation of rod photoreceptor inner segment with zoomed in panels showing two mid and a proximal region. The ER (false colored in *yellow*) can be seen running along the rootlet and in contact with mitochondria (*top panel*) and Golgi (*bottom panel*). (**C**) Serial block face scanning electron microscopy (SBFSEM) image slice of mouse retina that includes three inner segments that have been segmented (PM in *purple*, mitochondria in *blue*, and ER in *yellow*). (**D–F**) Subsequent rendered reconstruction generated from the segmented SBFSEM data. **D** The three segmented inner segments together and separated to show individual photoreceptors in **E**. **F** Clipped images showing the structure and position of the ER (*yellow*), mitochondria (*blue*), and Golgi (*red*) within the mid and proximal regions of the inner segment. *Scale bars* = **A** 200 nm and **B** 1 µm.

In addition to the ER associating with the rootlet, there are clear MCS between ER and both mitochondria and the plasma membrane within the inner segment (Fig. 4A). These types of MCS have been well studied in other cell types, and are frequently observed when examining electron microscopy data from the inner segment. When entire inner segments were imaged, the extensive association of ER with the rootlet and MCS with mitochondria, Golgi, and plasma membrane were evident (Fig. 4B). By segmenting the ER, mitochondria, and Golgi within serial block face scanning electron microscopy (SBFSEM) data it is possible to view these interactions in 3D throughout entire inner segments (Figs. 4C–F; Supplementary Videos S1–S6). Unfortunately, at the resolution of SBFSEM, it is not possible to resolve the rootlet, so this is not included in the segmentation. In the 3D models, the ER predominantly occupied the center of the inner segment in the distal part of the inner segments (see Figs. 4D–F), running in between and in contact with mitochondria (see Fig. 4F top panel). By contrast, within the proximal part of the inner segment the ER occupies a larger area and surrounded the Golgi apparatus (see Figs. 4D–F).

### Photoreceptor Rootlets Are in Contact With Both ER and Mitochondria in Human Rods

**Figure 5.**
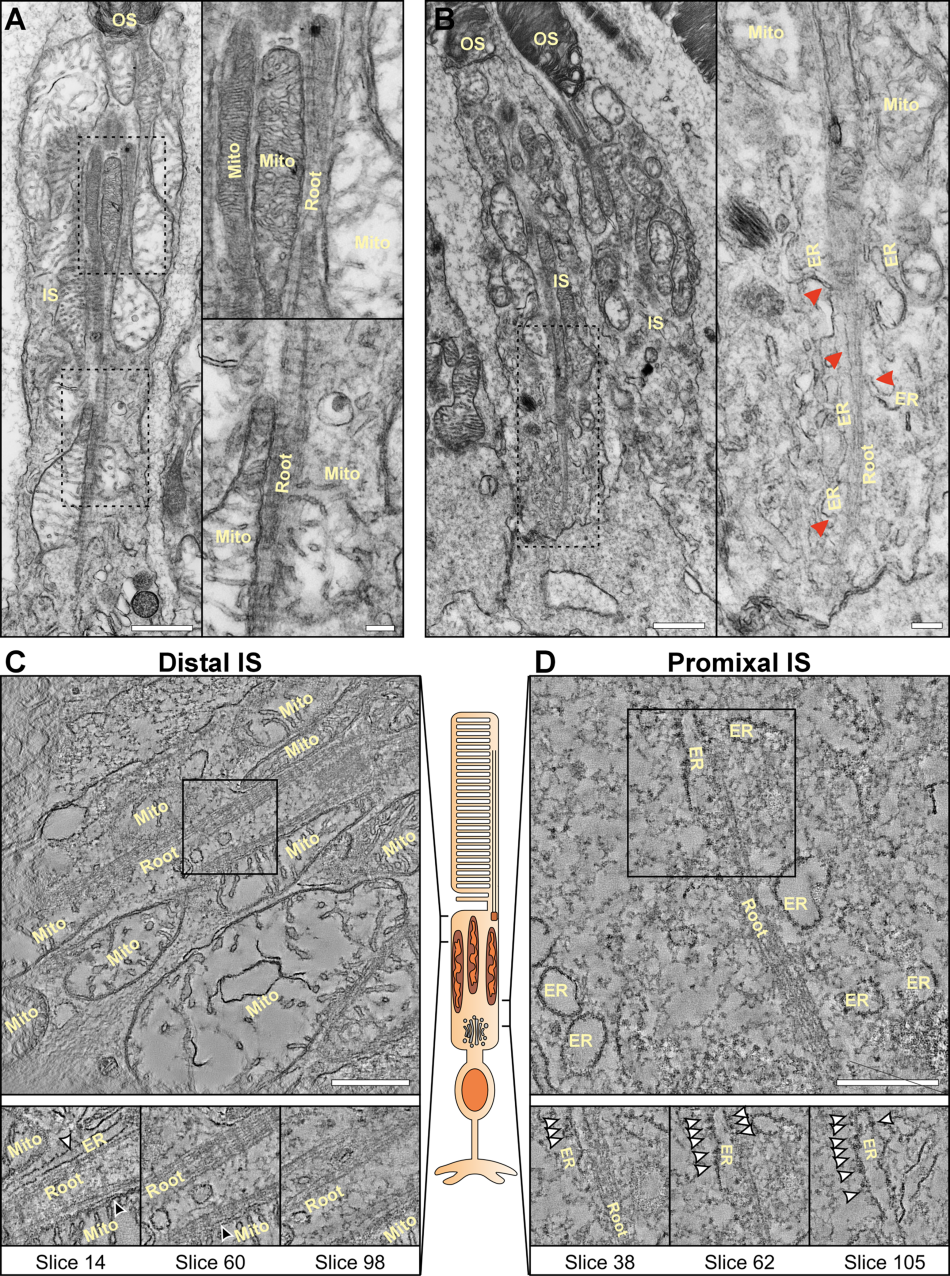
**Human rod photoreceptor rootlets contact both mitochondria and ER**. (**A,** **B**) Electron microscopy images of human rod photoreceptor within human retina. **A** At the distal and mid inner segment, the rootlet is associated with mitochondria. **B** Within the proximal region of inner segments, the ER can be seen running alongside the rootlet (Root) as denoted by the *red arrowheads*. (**C,** **D**) Tomograms of rod photoreceptors **C** within the distal inner segment, the rootlet is in contact mainly with mitochondria (*black arrowheads*) with some contact with the ER (*white arrowheads*). **D** In the proximal inner segment, there are less mitochondria than the distal region and ER is in contact with the rootlet. Ribosomes can be seen on the ER highlighted by the *white arrowheads* **D**. *Scale bars* = **A** and **B** *left panels* – 1 µm and **A** and **B** *right panels* = 200 nm and **C** and **D** = 500 nm.

To investigate if similar associations between ER and rootlet exist in human photoreceptors, human eye tissue was examined by electron tomography (Fig. 5; see Supplementary Fig. S2). In the proximal inner segment that has a low density of mitochondria, the rootlet associated with the ER, as in mouse inner segments (see Figs. 5B, 5D). Unlike mouse rod photoreceptor inner segments, the distal inner segments of human rods are densely packed with mitochondria (see Figs. 5A, 5C). When examining the distal inner segment of human photoreceptors, the rootlet was found to have some interaction with the ER but was more extensively in contact with mitochondria (see Fig. 5C).

## Discussion

In this study, we demonstrate that the photoreceptor ER has extensive interactions with the connecting cilium rootlet of mouse and to a lesser extent human rod inner segments. It appears that the ER uses the rootlet as a “scaffold,” where it is able to branch and make MCS with the mitochondria, Golgi, and plasma membrane. These are important findings, as photoreceptors are among the most energy-consuming cells within the body and demand extensive protein synthesis. It is likely that MCS assists in achieving these demands through intra-organelle communication.

Membrane structures in contact with the connecting cilium rootlet have been previously described. This includes both TEM and cryo-tomography studies investigating the role and structure of the rootlet.^4^^,^^22^ Using a range of TEM, immunolabeling, and mass spectrometry methods, we provide evidence that these membranes are ER and that the interactions with the rootlet are extensive running along the entire length of the inner segment in mouse photoreceptors. The rootlet was only examined in the inner segment and the interaction with the ER may extend further. This may explain why the connecting cilium rootlet is exceptionally long in photoreceptors compared with other ciliated cell types.^18^^,^^36^

For the ER to be associated with the rootlet, there is potentially some kind of attachment or tethering complex between these organelles. Membrane associated proteins were reported by Hoorn and Carter (2023) in cryo-tomography data that were described as globular membrane-associated densities approximately 7 nm in diameter that cover the membrane, which we have identified as ER.^22^ In addition to bundles of filaments running the length of the rootlet, they were able to resolve perpendicular cross-striations that were termed amorphous bands running the width of the rootlet. These amorphous bands were shown to come into direct contact with membrane or interact with the membrane associate proteins. Therefore, these structures could potentially act as tethers attaching the ER to the rootlet.

The P23H rhodopsin mouse model provides further evidence of the association and contact sites of the ER with the rootlet and mitochondria. In this model, the misfolded P23H rhodopsin is retained in the ER after its synthesis prior to degradation.^9^^,^^10^ By examining a heterozygous model at a timepoint with a fully developed retina and without complete photoreceptor cell loss (6 weeks), the localization of P23H rhodopsin to the ER surrounding the rootlet was revealed.

Homozygous P23H mice were used to interrogate the P23H interactome in order to avoid misinterpretation arising from WT rhodopsin present in the heterozygous model. Due to the rapid degeneration of P23H rhodopsin homozygous photoreceptors, we selected an early P10 timepoint.^37^ This was advantageous, as photoreceptors at P10 lack mature outer segments allowing a greater focus on inner segment proteins. This is supported by a previous study showing lack of a mature outer segment at P10 WT mice,^38^ and the absence of many outer segment proteins in our P10 WT mouse interactome data. The immunopurification of P23H rhodopsin homozygous mice led to a significant enrichment of rootletin compared to control WT mice, where it was almost undetectable. Mitochondrial proteins and ER folding factors and quality control proteins were also enriched in the P23H retina, but to a lesser extent than rootletin. This supports the existence of ER:mitochondrial MCS in photoreceptors and reveals how extensive the interactions are between the ER and rootlet. It should be considered, however, that P23H as a misfolded aggregation prone protein could form nonspecific interactions with other protein complexes during the immunopurification. To reduce the potential for purification of nonspecific binding proteins, we used a detergent and magnetic bead immunopurification approach with highly stringent 1D4 peptide elution that has been used to study rhodopsin structure and function. In addition, the majority of the co-purifying proteins were previously identified in a similar study of WT and P23H rhodopsin interactomes.^32^ Nevertheless, several of the co-purifying proteins are commonly found in the “crapome” highlighting potential nonspecific interactions. Although rootletin is not a common component in other interactome datasets, the abundance of rootletin and size of the rootlet in photoreceptors could increase the chance of nonspecific interactions between aggregation prone P23H and rootletin during lysis and purification. Therefore, the association of rootletin with P23H rhodopsin could reflect the physical proximity of the proteins, because P23H is retained in the ER and/or nonspecific interactions during purification, rather than a direct functional interaction.

Previously, peripherin/rds was reported to be associated with the rootlet in degenerating photoreceptors with the protein described as being present in vesicles adjacent to the rootlet.^20^ This led to the authors’ hypothesis that the rootlet could act as a transporter. It is more likely that these vesicles are ER that consists of accumulated protein that is unable to correctly traffic to the outer segment and the rootlet is not a transporter.

The shortening of outer segments and eventual cell death observed in rootletin mutant mice that lack rootlets,^21^ could be linked to alterations in ER membrane contact sites. The shortening of outer segments indicates a trafficking defect and that components required to generate discs are not being transported efficiently enough. It was previously reported in the rootletin mutant mice that they had randomly dispersed membranous organelles within the inner segment, which we show here are likely to be ER.^21^ The absence of the rootlet to help position and guide the ER throughout the inner segment could affect ER shape and MCS with other organelles. As a result, this could impact ER function, including protein synthesis and signaling. Energy production could also be indirectly affected by dysregulated MCS between ER and mitochondria.^39^^,^^40^ When ER morphology is affected, such as in cases with pathogenic mutations in *REEP6.1* that encodes an ER shaping protein, there is sight loss as a result of retinitis pigmentosa.^34^^,^^41^

Mice have mitochondria positioned against the plasma membrane leaving the center of the inner segment devoid of mitochondria.^29^ In contrast, human photoreceptors have a higher density of mitochondria throughout the distal inner segment, leaving little room for other organelles. This coincided with the distal region of human rod photoreceptors inner segments having mainly mitochondria in contact with the rootlet. Similar interactions between rootlets and mitochondria have been previously documented in the *amphioxus lanceolatus* endostyle.^42^ The proximal region of the inner segment was more like the mouse, with extensive interactions between the rootlet and ER. With the greater packing of mitochondria in human distal inner segment, it is likely that the ER runs between and makes contact with mitochondria without the need of the rootlet as a “scaffold.” This difference in mitochondrial density, ER shape, and rootlet contact sites between humans and mice is particularly relevant when considering certain transgenic mice models for human disease.

The data reported here has uncovered the interaction of the ER with the rootlet and shows important distinctions between the MCS found in mouse and human rod photoreceptors. To better understand the role of the rootlet in positioning and shaping the ER further studies are needed. Our finding may provide new insight into photoreceptor disease, as factors that impact interactions between the ER and rootlet could have an impact on ER MCS, as well as affecting membrane and protein trafficking.

## Supplementary Material

Supplement 1

Supplement 2

Supplement 3

Supplement 4

Supplement 5

Supplement 6

Supplement 7

Supplement 8
