# Supplementary material for: The P23H Rhodopsin Mouse Model Reveals a Novel Interaction Between the Endoplasmic Reticulum and Connecting Cilium Rootlet Within Photoreceptors
Source: Invest Ophthalmol Vis Sci. 2026 Mar 30;67(3):57. doi: 10.1167/iovs.67.3.57 (PMC13037744; doi:10.1167/iovs.67.3.57)
Supplement: Supplement 2 [file iovs-67-3-57_s002.docx]

The P23H rhodopsin mouse model reveals a novel interaction between the endoplasmic reticulum and connecting cilium rootlet within photoreceptors

**Table S1** is available as a excel file.

| **Protein names** | **Gene name** | **Organelle and/or function** | **WT 1D4 elution peptides measured** | **P23H 1D4 elution peptides measured** | **Fold enrichment in P23H** |
| --- | --- | --- | --- | --- | --- |
| **Heat shock cognate 71 kDa protein** | **Hspa8** | Chaperone | 3 (± 7.78) | 14 (± 0.00) | 4.7 |
| **Protein sel-1 homolog 1** | **Sel1l** | ER | 0.5 (± 2.83) | 5 (± 1.41) | 10.0 |
| **Heat shock protein HSP 90-alpha** | **Hsp90aa1** | Chaperone | 1.5 (± 2.83) | 5 (± 1.41) | 3.3 |
| **Ubiquitin-ribosomal protein eS31 fusion protein** | **Rps27a** | Cytoplasm | 3 (± 0.35) | 3.5 (± 0.71) | 1.2 |
| **Protein OS-9** | **Os9** | ER | 0 (± 2.12) | 3 (± 0.00) | P23H only |
| **60 kDa heat shock protein** | **Hspd1** | Chaperone | 0 (± 2.12) | 3 (± 1.41) | P23H only |
| **Heat shock protein HSP 90-beta** | **Hsp90ab1** | Chaperone | 1 (± 1.77) | 2.5 (± 0.71) | 2.5 |

**Table S2. Chaperones and ER quality control components enriched in the P23H interactome**. Proteins immunopurified and eluted with 1D4 peptide from P10 WT and P23H rhodopsin homozygous mouse retina. Mean ± SD of spectral counts measured from two independent purifications. Proteins in bold were also identified in proteomics data of a previous study of P23H rhodopsin homozygous mouse retina.^1^ Two samples were examined, and these were formed from four pooled eyes per genotype.

| **Protein names** | **Gene name** | **WT 1D4 elution peptides measured** | **P23H 1D4 elution peptides measured** | **Fold enrichment in P23H** |
| --- | --- | --- | --- | --- |
| **Prohibitin 1** | **Phb1** | 2.5 (± 4.60) | 9.5 (± 0.71) | 3.8 |
| **Non-selective voltage-gated ion channel VDAC1** | **Vdac1** | 2.5 (± 4.24) | 8 (± 1.41) | 3.2 |
| **ATP synthase F(1) complex subunit alpha** | **Atp5f1a** | 17 (± 5.66) | 8 (± 0.00) | 0.5 |
| **ATP synthase F(1) complex catalytic subunit beta** | **Atp5f1b** | 26.5 (± 13.44) | 8 (± 1.41) | 0.3 |
| **ADP/ATP translocase 2** | **Slc25a5** | 4 (± 2.83) | 6 (± 2.83) | 1.5 |
| **ADP/ATP translocase 1** | **Slc25a4** | 4 (± 1.77) | 5.5 (± 0.71) | 1.4 |
| **Cytochrome b-c1 complex subunit 2** | **Uqcrc2** | 1 (± 3.18) | 5.5 (± 2.12) | 5.5 |
| **Prohibitin 2** | **Phb2** | 0 (± 3.18) | 4.5 (± 0.71) | P23H only |
| **Solute Carrier Family 25 Member 3** | **Slc25a3** | 2.5 (± 1.41) | 4 (± 1.41) |  |
| **ATP synthase peripheral stalk subunit OSCP** | **Atp5po** | 4 (± 0.71) | 3 (± 0.00) | 0.8 |
| NADH-ubiquinone oxidoreductase 75 kDa subunit | Ndufs1 | 0 (± 2.12) | 3 (± 1.41) | P23H only |
| **Non-selective voltage-gated ion channel VDAC2** | **Vdac2** | 1 (± 1.77) | 2.5 (± 0.71) | 2.5 |
| **MICOS complex subunit Mic60** | **Immt** | 0 (± 1.41) | 2 (± 1.41) | P23H only |

**Table S3. Mitochondrial components enriched in the P23H interactome**. Proteins immunopurified and eluted with 1D4 peptide from P10 WT and P23H rhodopsin homozygous mouse retina. Mean ± SD of spectral counts measured from two independent purifications. Proteins in bold were also identified in proteomics data of a previous study of P23H rhodopsin homozygous mouse retina.^1^ Two samples were examined, and these were formed from four pooled eyes per genotype.

| **Protein names** | **Gene name** | **WT 1D4 elution peptides measured** | **P23H 1D4 elution peptides measured** | **Fold enrichment in P23H** |
| --- | --- | --- | --- | --- |
| Large ribosomal subunit protein eL43 | Rpl37a | 3 (± 2.47) | 4.5 (± 3.54) | 1.5 |
| Small ribosomal subunit protein eS19 | Rps19 | 0 (± 2.12) | 3 (± 2.83) | P23H only |
| **Large ribosomal subunit protein eL13** | **Rpl13** | 0 (± 2.12) | 3 (± 0.00) | P23H only |
| **Small ribosomal subunit protein uS3** | **Rps3** | 1.5 (± 0.35) | 2.5 (± 0.71) | 1.7 |
| **Small ribosomal subunit protein uS11** | **Rps14** | 0 (± 1.77) | 2.5 (± 0.71) | P23H only |
| **Large ribosomal subunit protein uL11** | **Rpl12** | 0 (± 1.77) | 2.5 (± 0.71) | P23H only |
| **Small ribosomal subunit protein eS25** | **Rps25** | 0 (± 1.41) | 2 (± 1.41) | P23H only |
| **Large ribosomal subunit protein uL14** | **Rpl23** | 0.5 (± 1.41) | 2 (± 2.83) | 4.0 |
| **Small ribosomal subunit protein eS4** | **Rps4x** | 0 (± 1.41) | 2 (± 1.41) | P23H only |

**Table S4. Ribosomal proteins enriched in the P23H interactome**. Proteins immunopurified and eluted with 1D4 peptide from P10 WT and P23H rhodopsin homozygous mouse retina. Mean ± SD of spectral counts measured from two independent purifications. Proteins in bold were also identified in proteomics data of a previous study of P23H rhodopsin homozygous mouse retina.^1^ Two samples were examined, and these were formed from four pooled eyes per genotype.

| **Protein names** | **Gene name** | **WT 1D4 elution peptides measured** | **P23H 1D4 elution peptides measured** | **Fold enrichment in P23H** |
| --- | --- | --- | --- | --- |
| Rootletin | Crocc | 0.5 (± 43.13) | 62 (± 0.00) | 124.0 |
| **Tubulin beta-5 chain** | **Tubb5** | 42 (± 6.72) | 51.5 (± 0.71) | 1.2 |
| Tubulin alpha-1A chain | Tuba1a | 35.5 (± 7.42) | 42.5 (± 4.95) | 1.2 |
| **Actin, alpha skeletal muscle** | **Acta1** | 3.5 (± 13.44) | 23 (± 0.00) | 6.6 |
| Vimentin | Vim | 42 (± 11.67) | 21.5 (± 0.71) | 0.5 |
| **Spectrin alpha chain, non-erythrocytic 1** | **Sptan1** | 0 (± 14.14) | 20 (± 4.24) | P23H only |
| Actinin Alpha 4 | Actn4 | 0.5 (± 13.08) | 19.5 (± 0.71) | 39.0 |
| **Myosin-10** | **Myh10** | 1 (± 10.61) | 16 (± 1.41) | 16.0 |
| Drebrin | Dbn1 | 0.5 (± 10.25) | 14.5 (± 3.54) | 29.0 |
| Myosin-9 | Myh9 | 2 (± 9.90) | 14 (± 7.07) | 7.0 |
| F-actin-capping protein subunit alpha-2 | Capza2 | 1.5 (± 7.78) | 11 (± 1.41) | 7.3 |
| **Tubulin beta-2A chain** | **Tubb2a** | 8.5 (± 1.06) | 10.5 (± 2.12) | 1.2 |
| **Spectrin beta chain, non-erythrocytic 1** | **Sptbn1** | 0 (± 6.36) | 9 (± 1.41) | P23H only |
| **Tubulin beta-4B chain** | **Tubb4b** | 5.5 (± 1.41) | 8 (± 0.00) | 1.5 |
| Junction plakoglobin | Jup | 12 (± 2.83) | 8 (± 1.41) | 0.7 |
| Desmoplakin | Dsp | 16.5 (± 7.78) | 7 (± 0.00) | 0.4 |
| F-actin-capping protein subunit alpha-1 | Capza1 | 0 (± 4.60) | 6.5 (± 0.71) | P23H only |
| F-actin-capping protein subunit beta | Capzb | 0 (± 4.24) | 6 (± 0.00) | P23H only |
| Tropomodulin-2 | Tmod2 | 0 (± 3.89) | 5.5 (± 0.71) | P23H only |
| Myosin light polypeptide 6 | Myl6 | 0 (± 2.83) | 4 (± 0.00) | P23H only |
| **Tubulin beta-4A chain** | **Tubb4a** | 3 (± 1.06) | 3.5 (± 2.12) | 1.2 |
| **Keratin, type II cytoskeletal 2 oral** | **Krt76** | 4 (± 0.35) | 3.5 (± 0.71) | 0.9 |
| **Lamin B1** | **Lmnb1** | 0 (± 2.12) | 3 (± 0.00) | P23H only |
| Alpha-actinin-1 | Actn1 | 0 (± 1.77) | 2.5 (± 2.12) | P23H only |
| **Cofilin 1** | **Cfl1** | 1.5 (± 0.35) | 2.5 (± 0.71) | 1.7 |
| **Unconventional myosin-VI** | **Myo6** | 0 (± 1.77) | 2.5 (± 0.71) | P23H only |
| Tropomyosin 1 | Tpm1 | 1 (± 1.06) | 2.5 (± 2.12) | 2.5 |
| Tropomodulin 3 | Tmod3 | 0 (± 1.41) | 2 (± 1.41) | P23H only |

**Table S5. Cytoskeleton components enriched in the P23H interactome**. Proteins immunopurified and eluted with 1D4 peptide from P10 WT and P23H rhodopsin homozygous mouse retina. Mean ± SD of spectral counts measured from two independent purifications. Proteins in bold were also identified in proteomics data of a previous study of P23H rhodopsin homozygous mouse retina.^1^ Two samples were examined, and these were formed from four pooled eyes per genotype.


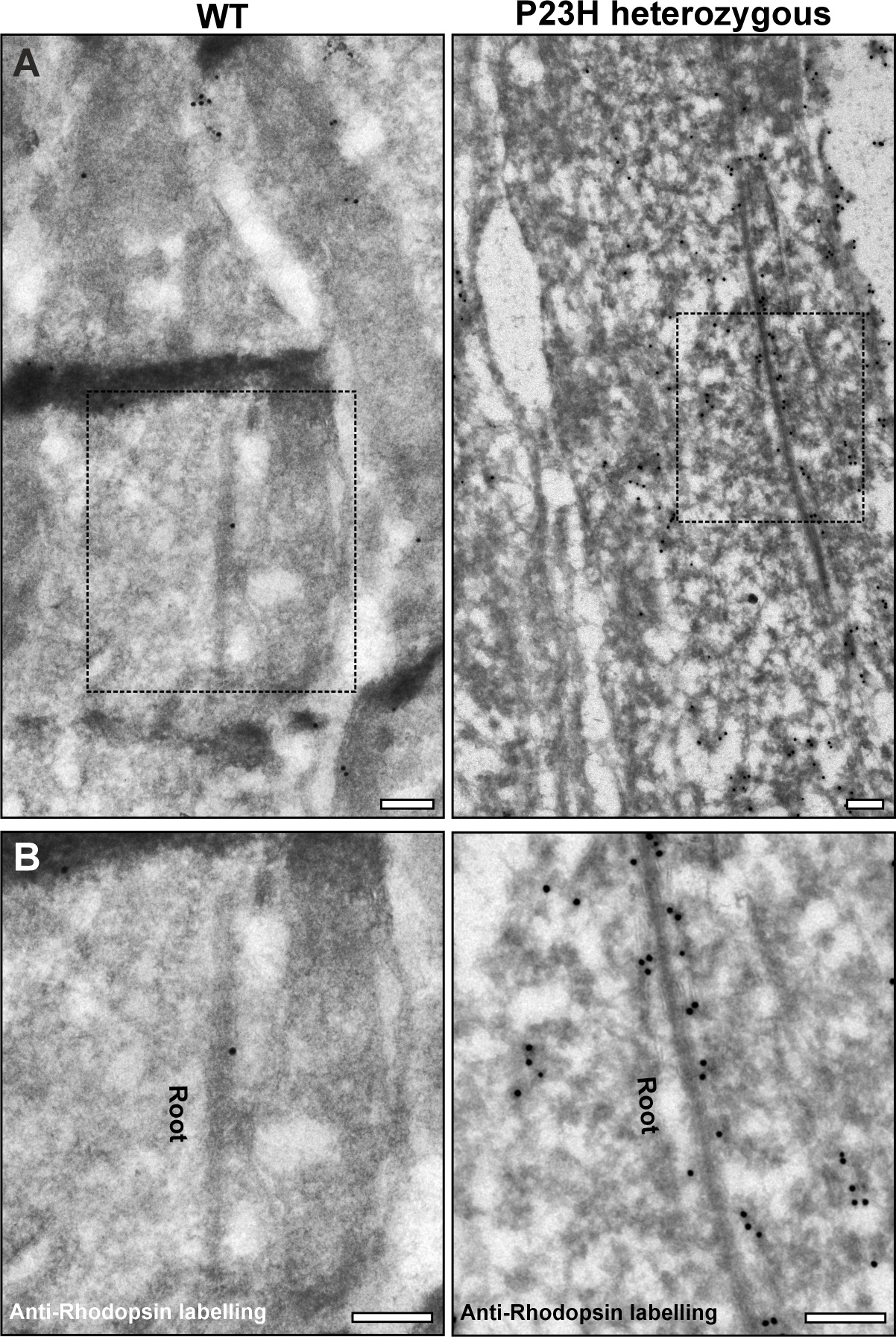


**Figure S1.** Further examples of rhodopsin localised at the photoreceptor rootlet of 6-week-old P23H heterozygous mice. (A - B) ImmunoEM labelling for rhodopsin in 6-week-old WT and heterozygous P23H mouse retina with (B) zoomed in region focusing on the rootlet. (B) Small amount of rhodopsin immunoEM labelling found in WT mouse compared to heterozygous P23H mouse retina, where much more staining is close to the rootlet. Scale bar: 200nm.


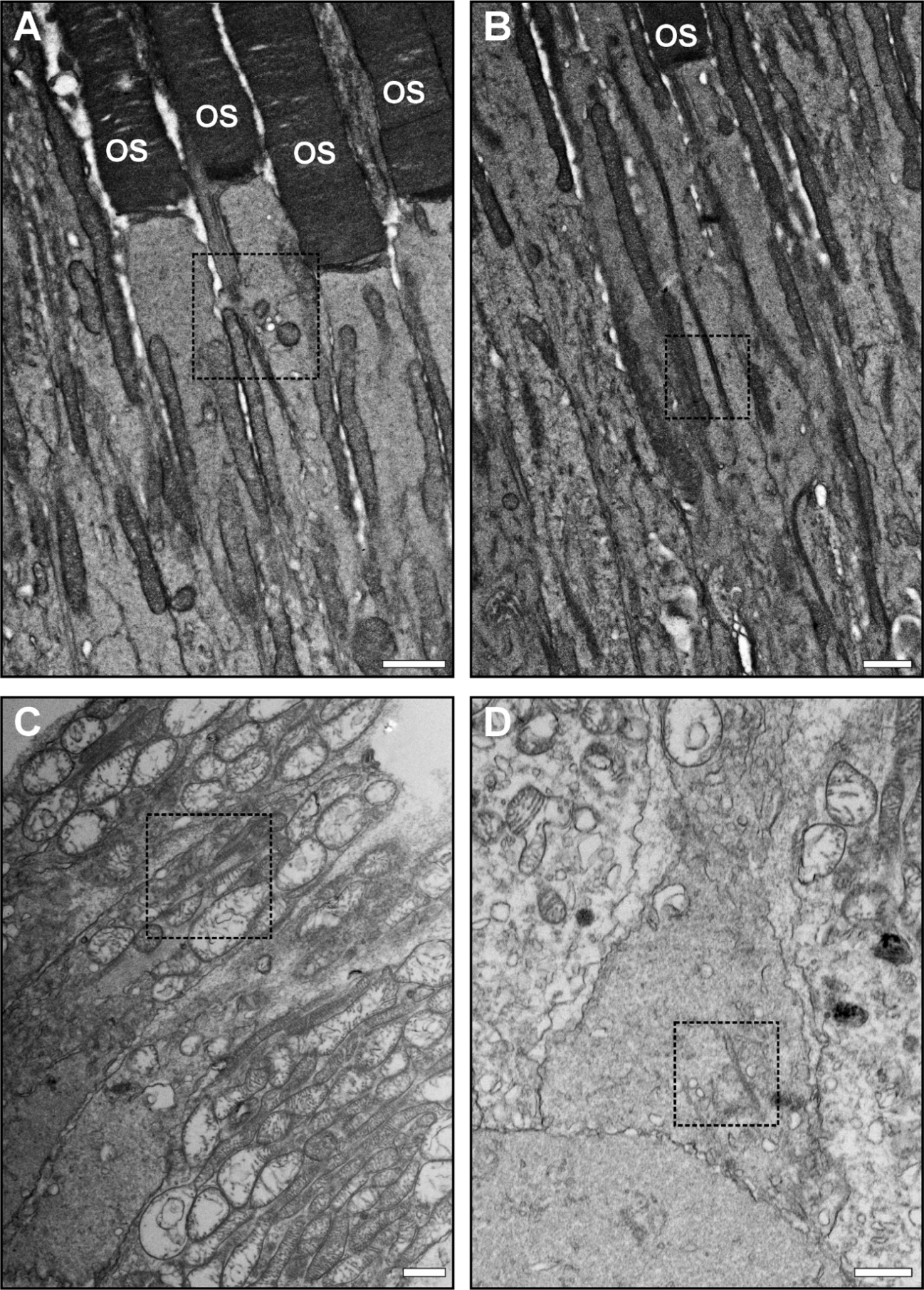


**Figure S2**. Low magnification electron microscopy images of the regions selected for electron tomography analysis. (A & B) Photoreceptors of control mouse retina with boxes highlighting the areas used to generate tomograms in Figure 2. (C & D) Photoreceptors of human retina with boxes showing the regions used to make tomograms in Figure 5. Scale bar: 1µm.


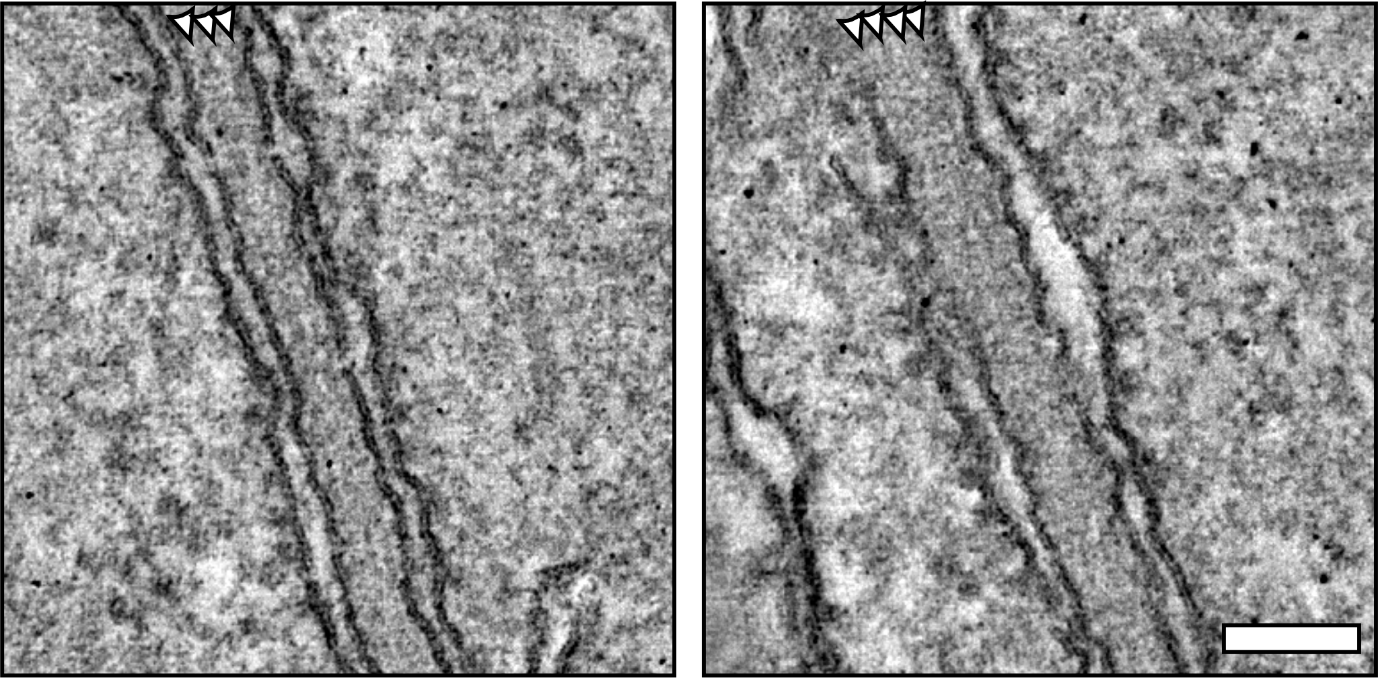


**Figure S3**. The filamentous content of the rootlet can be seen within electron tomography data. Images of control mice photoreceptors showing a projection of 10 tomogram slices for the two tomograms shown in Figure 2. Filaments within the rootlets can be seen as denoted by the white arrowheads. Scale bar: 100mn.


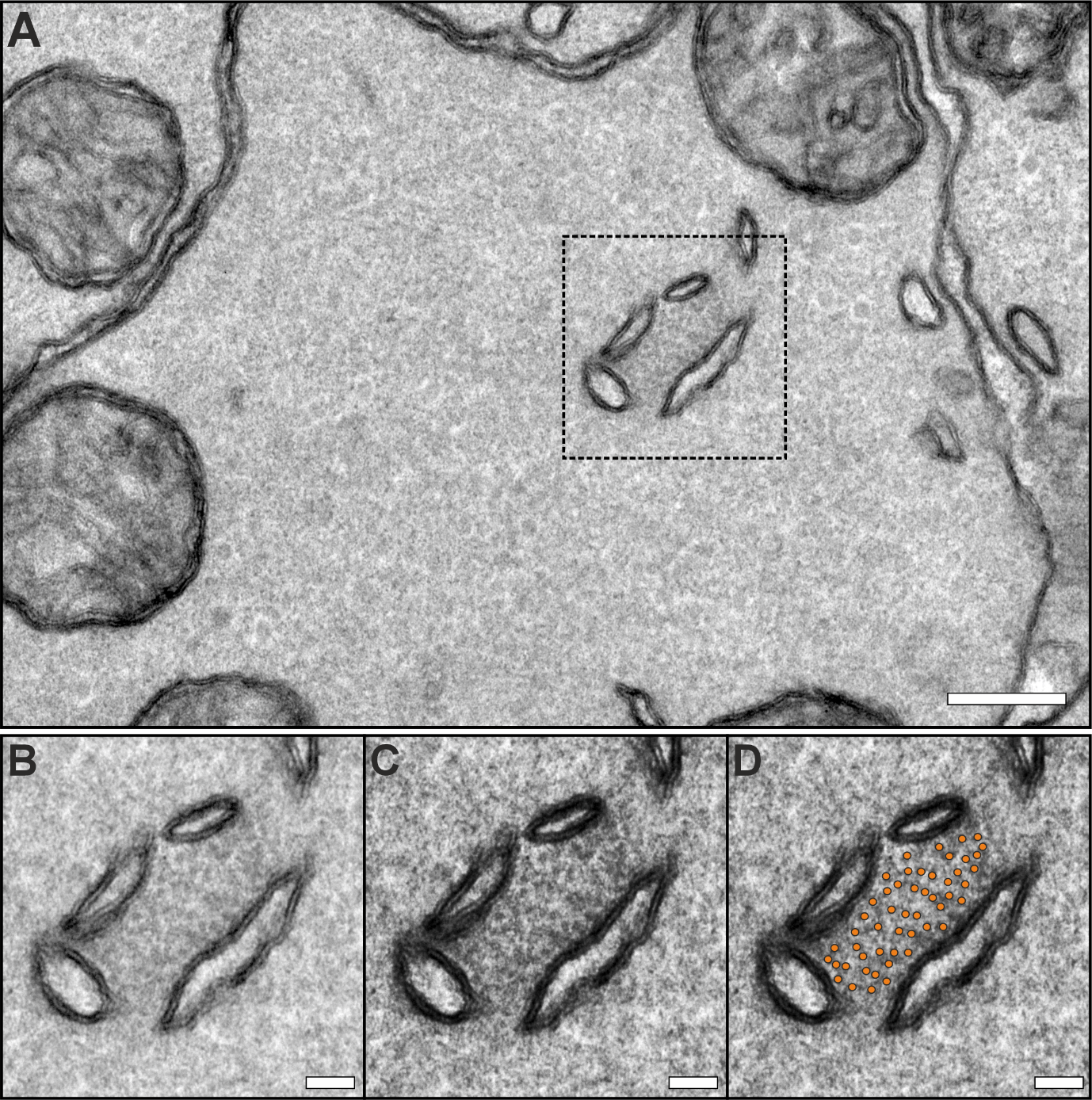


**Figure S4**. Identification of a rootlet from a cross-sectionally orientated sample by the evidence of filaments running in plane of view. (A-D) Control mouse photoreceptor orientated cross sectionally which is shown in Figure 2. The boxed region in (A) is shown at a higher magnification in (B-D). (C) is a contrast enhanced version of (B), allowing the filament running in the plane of view to be more easily resolved. (D) The filaments are false coloured in orange to highlight their position. Scale bar: (A) 200nm and (B-D) 50nm.


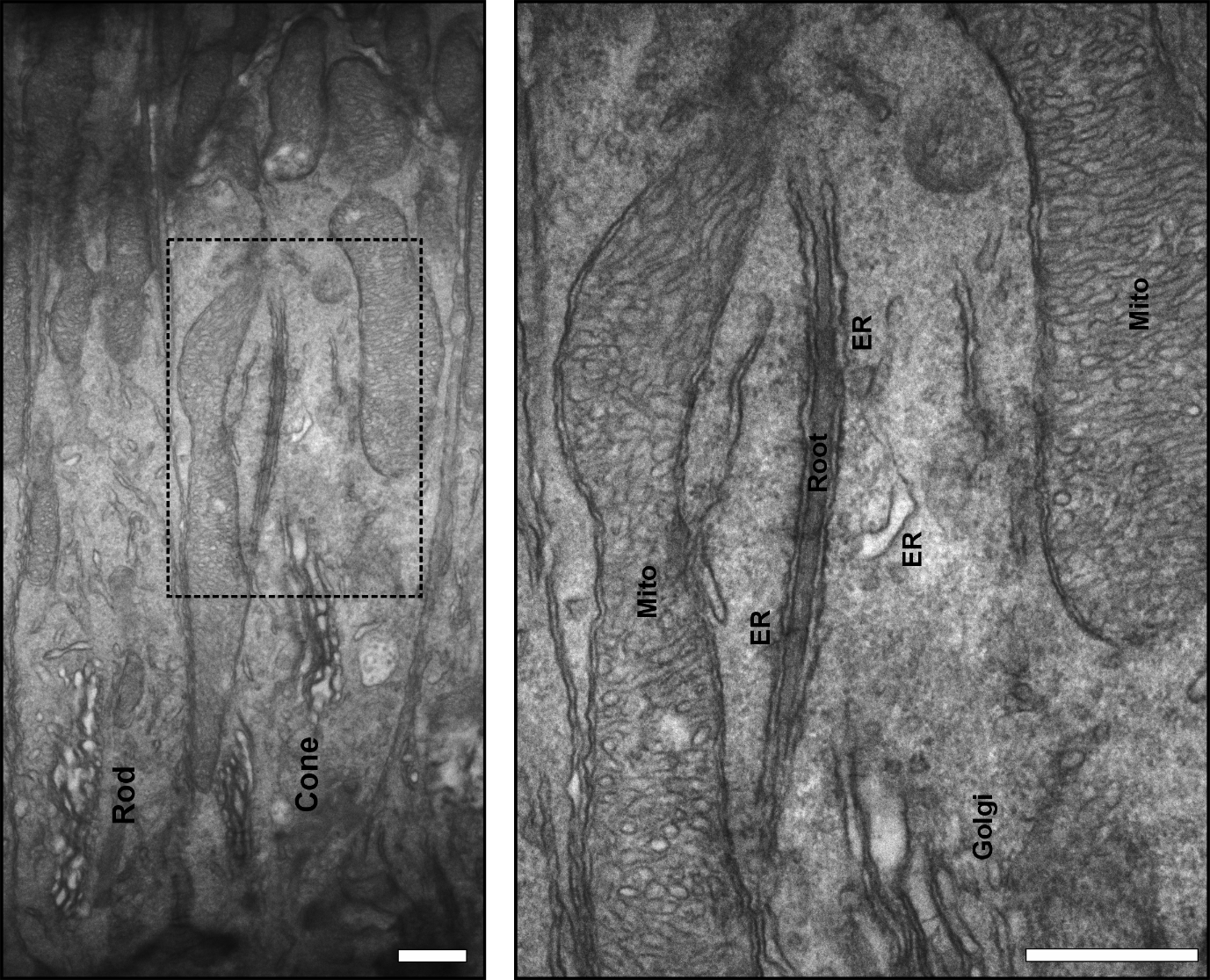


**Figure S5**. Membranes associated with the cone rootlet. Similar to the observations from rod photoreceptors shown in Figure 2, ER like membranes can be seen associated with the connecting cilium rootlet within the cone inner segment. Cones can be distinguished from rods by electron microscopy due to the wider inner segment and larger mitochondria. Scale bar: 500mn.


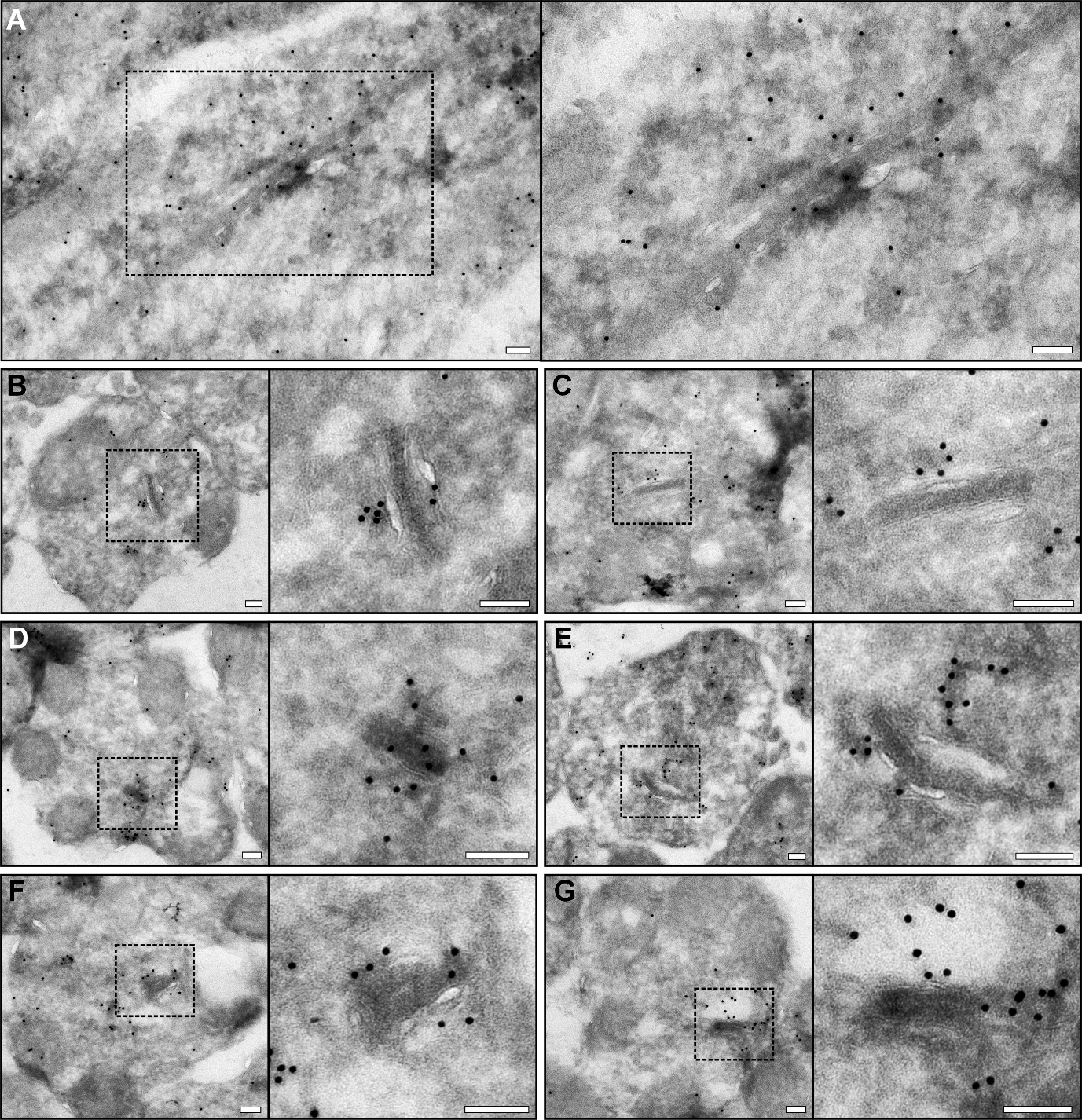


**Figure S6**. Further examples of anti-KDEL immuno electron microscopy staining. (A) longitudinally and (C - G) cross-sectionally orientated control mouse retina with immuno gold staining for the ER protein KDEL. (A - G) The dotted box in the left panels is shown at higher magnification in the right panels. (B – G) Photoreceptor are slightly oblique leading to a small strip of the rootlet being visible. (B) Is the photoreceptor rootlet shown in Figure 3E. Scale bar: 100nm
